# Inorganic-Nanoparticle Modified Polymers

**DOI:** 10.3390/polym14101979

**Published:** 2022-05-12

**Authors:** Ana M. Díez-Pascual

**Affiliations:** Universidad de Alcalá, Facultad de Ciencias, Departamento de Química Analítica, Química Física e Ingeniería Química, Ctra. Madrid-Barcelona, Km. 33.6, 28805 Alcalá de Henares, Madrid, Spain; am.diez@uah.es; Tel.: +34-918-856-430

Inorganic nanoparticle-modified polymer nanocomposites have attracted substantial attention over the last years in the preparation of materials for a number of applications. Numerous types of inorganic nanoparticles, such as quantum dots, metal oxides, mesoporous silica, gold and silver nanoparticles, and ceramics (Figure 1) have been incorporated into polymers for application in a wide range of fields, such as medicine, photovoltaics, packaging, and structural applications, etc.

One of the major aims of the addition of inorganic nanoparticles to polymers is to enhance their mechanical and thermal properties. Thus, a few percent of clay usually confers improved flame retardant behavior, higher stiffness and strength, and enhanced barrier properties against gases and vapors compared to the neat polymer [1,2,3,4]. Nanoparticles display a higher specific surface area compared to microsized particles and can therefore lead to better properties than those attained using conventional microfiller loadings. However, a number of tasks have to be completed first. Nanoparticle agglomeration within the polymer matrix and poor interfacial adhesion between the nanofillers and the matrix often impede property improvements, which represent a challenge in modifying the surface chemistry to promote physical or chemical interactions with polymer chains. The final properties of nanocomposites depend directly on several factors such as the chemistry of the polymer matrix, the filler-polymer affinity, the geometry of the particle, its degree of orientation and dispersion inside the polymer, as well as the means of nanocomposite preparation. This Special Issue, with a collection of seven original contributions, provides selected examples of the most recent advances in the preparation, characterization, and application of polymeric nanocomposites comprising nanoparticles.

Over the last years, inorganic nanotubes, fullerenes, and 2D nanomaterials based on layered metal dichalcogenides such as WS_2_ and MoS_2_, have arisen as very promising nanostructures over their carbon counterparts owed to their low toxicity and biocompatibility, enabling their use for environmental and biomedical applications [5]. Numerous research works have investigated the promising tribological, mechanical, and barrier properties of WS_2_ nanomaterials, making them an excellent alternative to carbon nanotubes and graphene as nanofillers in polymeric nanocomposites [6,7,8,9]. Besides, WS_2_ nanoparticles demonstrate great potential for mechanical reinforcement of a variety of biopolymers [10,11,12], showing good manufacturability and performance, as well as reduced manufacturing costs compared to other inorganic or organic nanomaterials such as nanoclays, carbon nanotubes, etc.

These types of nanoparticles are currently the subject of intense research. In particular, inorganic fullerene (IF)-like WS_2_ nanoparticles ranging from 0.1 to 1 wt% were integrated into a poly(L-lactic acid) (PLLA) matrix via a simple melt extrusion process [13]. TEM and SEM results revealed that the nanoparticles were well dispersed in the PLLA matrix without the need for a compatibilizer or modifier. DSC analysis showed that the crystallization and melting temperature as well as the crystallinity of PLLA were controlled by the cooling rate and composition. The crystallization behavior and kinetics were examined using the Lui model, and the nucleating effect of the nanoparticles was assessed based on Gutzow and Dobreva approaches. It was found that the incorporation of increasing IF-WS_2_ contents speeds up the crystallization rate of PLLA. This high performance of these novel bionanocomposites could be suitable for different industrial applications.

The same authors used tungsten disulfide nanosheets (2D-WS2) as nanofillers for PLLA, in the same concentration range [14]. In this case, the analysis of the cold-crystallization behavior of the PLLA showed that the crystallization rate of the polymer was reduced upon nanosheet incorporation. Unpredictably for polymer nanocomposites, a radical change from retardation to promotion of crystallization was found upon raising the nanosheet content, whereas the melt-crystallization mechanism of PLLA remained unchanged. On the other hand, the double-melting peaks, mainly derived from melting–recrystallization–melting processes upon heating, and their dynamic behavior were consistent with the effect of 2D-WS2 involved in the crystallization of PLLA. 

IF-WS_2_ nanoparticles in the range of 0.1–2 wt% have also been used as nanofillers for the manufacturing of polyethylene terephthalate (PET)/polypropylene (PP)-based nanocomposite blends via melt extrusion [15]. The nanocomposites were characterized by scanning electron microscopy (SEM), transmission electron microscopy (TEM), thermogravimetric analysis (TGA), dynamic differential scanning (DSC), dynamic mechanical analysis (DMA), and X-ray diffraction (XRD). The aim of the strategy was to increase the compatibility of PP and PET wherein IF-WS_2_ localized at the interface of the blend components. The tensile strength of the PP/PET increased by 32% with 1% IF-WS_2_, and the thermal stability by 18 °C with 2 wt%. This route can also be used for the large-scale production of such materials to be used in high-temperature environments.

On the other hand, conjugated polymers (CPs) are widely used in photovoltaic devices and organic light-emitting diodes (OLEDs) [16]. The alternating carbon–carbon double bonds in their structure are responsible for their electronic properties, low-energy optical transitions, and high-electron affinities that can be tuned either by blending compatible CPs or controlling the energy transfer between the CPs or a combination of both. For instance, a full-color emitter for white OLEDs (WOLEDs) can be achieved by blending different CPs that emit lights covering the entire visible spectrum. One widely explored binary blend WOLED is the combination of poly(9,9-dioctylfluorene-2,7-diyl) (PFO) and poly(2-methoxy-5(2-ethylhexyl)-1,4-phenylenevinylene (MEH-PPV) that produces blue and red emissions, respectively. In this blend, PFO with large energy band gap acts as a donor whereas MEH-PPV is the acceptor. Over the years, numerous efforts have been made to improve the luminance of this blend WOLED by either improving the charge carrier transportation via the modification of electrodes [17], or through blending with metal oxide nanoparticles [18]. In this regard, Al-Bati et al. [19] have investigated the effects of two acceptors on the emission characteristics of the PFO/F8BT/MEH-PPV ternary blend, and assessed the feasibility of TiO_2_ nanoparticles as quenching inhibitors as well as emission enhancers for the ternary blends. The effect of TiO_2_ nanoparticles on the surface morphology, photophysical properties, and current density–voltage (J-V) characteristics of the ternary blend thin films was also investigated, and the optimum amount of TiO_2_ nanoparticles was found to be 10 wt%.

The same authors also investigated the effect of SiO_2_/TiO_2_ nanocomposites on the properties of the PFO/MEH-PPV blend [20] using steady-state and time-resolved photoluminescence spectroscopies at room temperature. The improved energy transfer from PFO to MEH-PPV upon increasing nanocomposite content was demonstrated by examining absorbance, emission, and photoluminescence excitation spectra. The shorter values of the quantum yield and lifetime of the PFO in the hybrid thin films compared with the pure PFO indicate efficient energy transfer from PFO to MEH-PPV. The Förster radius was tuned from 44 to 76 Å, the distance between the molecules of donor and acceptor increased from 23 to 69 Å while the conjugation length slightly increased from −4.278 to −1.020 Å when the nanocomposite content increased from 0 to 20 wt%. Thus, the incorporation of SiO_2_/TiO_2_ nanocomposites into CPs plays a crucial role in the improvement of OLED devices’ performance.

Another conjugated conducting polymer, poly(3,4-ethylenedioxythiophene):poly(styrene sulfonate) (PEDOT:PSS) is widely used for practical applications such as energy conversion and storage devices, due to its good flexibility, processability, high electrical conductivity, and superior optical transparency, among others. Nonetheless, its hygroscopic character, short durability, and poor thermoelectric performance compared to inorganic counterparts have greatly limited its high-tech applications. In a recent work, SnO_2_ (0.5–10 wt%) was incorporated into PEDOT:PSS via a simple, low-cost, environmentally friendly method without the need for organic solvents or compatibilizing agents [21]. The morphology, thermal, thermoelectrical, optical, and mechanical properties of the resulting nanocomposites were extensively explored. A uniform dispersion of the SnO_2_ nanoparticles within the matrix was revealed by SEM microscopy, and the Raman spectra revealed the existence of very strong SnO_2_-PEDOT:PSS interactions. The stiffness and strength of the matrix steadily improved with increasing SnO_2_ content, up to 120% and 65%, respectively, upon the addition of 10 wt% SnO_2_. Furthermore, the nanocomposites showed greater thermal stability (by up to 70 °C), improved electrical conductivity (up to 140%), and higher Seebeck coefficient (about 80% increase) than neat PEDOT:PSS, while the optical transparency remained almost unchanged. These sustainable nanocomposites display noticeably better performance compared to commercial PEDOT:PSS, and can be applied in energy storage, flexible electronics, thermoelectric devices, and so forth. 

Over the last decades, severe environmental concerns and food safety problems have led to the development and application of degradable active packaging materials [22,23]. Among the widely known degradable polymers, poly(vinyl alcohol) (PVA) is a synthetic, water-soluble polymer used in the packaging industry due to its excellent film-forming performance, oxygen barrier properties, and chemical resistance [24]. However, the poor barrier and antibacterial properties of PVA have limited its application in active food packaging [25]. To overcome these deficiencies, one of the most effective methods is to prepare nanocomposites by blending PVA matrix with functional nanoparticles. In this regard, layered double hydroxides (LDHs) are a class of layered anionic synthetic clay, which have high anion exchange capacity and chemical purity, uniform structure, and controllable chemical composition. Organophilic LDHs coated by TA-Ti coordination compounds (LDHs@TA-Ti) were prepared by a green and simple method and incorporated into PVA via a solution casting process [26]. The effect of LDHs@TA-Ti on the thermal, mechanical, barrier, and antibacterial properties of LDHs@TA-Ti/PVA nanocomposites was investigated. Upon TA-Ti coating onto the LDHs surface, the antibacterial rate of LDHs@TA-Ti was increased up to 99.98%, as a result of the synergistic effect from the phenolic hydroxyl groups and photocatalytic TiO_2_. Moreover, LDHs@TA-Ti/PVA nanocomposites displayed outstanding antibacterial properties. Compared with pure PVA, LDHs@TA-Ti/PVA nanocomposites showed a 41% increase in tensile strength, an 18% increase in elongation at break, a 36% reduction in oxygen permeability, and a diminution of 26% in water vapor permeability upon the addition of 1.0 wt% LDHs@TA-Ti. On the other hand, their UV transmittance decreased by 99% when LDHs@TA-Ti content reached 3 wt%. Therefore, these novel nanocomposites show a great potential in the active packaging application due to their outstanding mechanical, barrier and antibacterial properties.

## Figures and Tables

**Figure 1:**
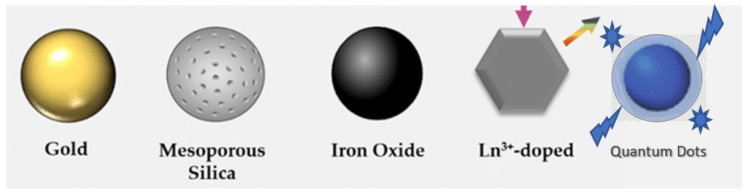
Commonly used inorganic nanoparticles to reinforced polymer matrices.
